# The DnaJ-like Zinc Finger Protein ORANGE Promotes Proline Biosynthesis in Drought-Stressed *Arabidopsis* Seedlings

**DOI:** 10.3390/ijms23073907

**Published:** 2022-03-31

**Authors:** Farman Ali, Qi Wang, Aliya Fazal, Lin-Juan Wang, Shuyan Song, Meng-Juan Kong, Tariq Mahmood, Shan Lu

**Affiliations:** 1 State Key Laboratory of Pharmaceutical Biotechnology, School of Life Sciences, Nanjing University, Nanjing 210023, China; alifarman978@gmail.com (F.A.); daisywang0908@163.com (Q.W.); aliyafaxal@gmail.com (A.F.); linjuan_w@163.com (L.-J.W.); maybessy@163.com (S.S.); 13260820993@163.com (M.-J.K.); 2 Department of Plant Sciences, Faculty of Biological Sciences Quaid-i-Azam University, Islamabad 45320, Pakistan; 3 Shenzhen Research Institute of Nanjing University, Shenzhen 518000, China

**Keywords:** drought, enzymatic activity, ORANGE, proline, Δ^1^-pyrroline-5-carboxylate synthase (P5CS)

## Abstract

Orange (OR) is a DnaJ-like zinc finger protein with both nuclear and plastidial localizations. OR, and its orthologs, are highly conserved in flowering plants, sharing a characteristic C-terminal tandem 4× repeats of the CxxCxxxG signature. It was reported to trigger chromoplast biogenesis, promote carotenoid accumulation in plastids of non-pigmented tissues, and repress chlorophyll biosynthesis and chloroplast biogenesis in the nucleus of de-etiolating cotyledons cells. Its ectopic overexpression was found to enhance plant resistance to abiotic stresses. Here, we report that the expression of OR in *Arabidopsis thaliana* was upregulated by drought treatment, and seedlings of the OR-overexpressing (OE) lines showed improved growth performance and survival rate under drought stress. Compared with the wild-type (WT) and OR-silencing (or) lines, drought-stressed OE seedlings possessed lower contents of reactive oxygen species (such as H_2_O_2_ and O_2_^−^), higher activities of both superoxide dismutase and catalase, and a higher level of proline content. Our enzymatic assay revealed a relatively higher activity of Δ^1^-pyrroline-5-carboxylate synthase (P5CS), a rate-limiting enzyme for proline biosynthesis, in drought-stressed OE seedlings, compared with the WT and or lines. We further demonstrated that the P5CS activity could be enhanced by supplementing exogenous OR in our in vitro assays. Taken together, our results indicated a novel contribution of OR to drought tolerance, through its impact on proline biosynthesis.

## 1. Introduction

Plants are constantly challenged by a variety of abiotic stresses, such as drought, high salinity, and extreme temperature, which highly reduce the yield of staple crops and are severe threats to worldwide food security [1]. For agricultural crop productivity, drought is one of the most frequent, widespread, and detrimental stresses [2]. Drought not only results in stomatal closure, which limits CO_2_ supply for photosynthesis, but also causes oxidative stresses by promoting the excessive production of reactive oxygen species (ROS) [3].

To cope with drought-induced oxidative stress, plants have developed various enzymatic and non-enzymatic protective mechanisms, such as the biosynthesis and accumulation of the low molecular weight-compatible compound proline [4,5,6]. It was demonstrated that the application of exogenous proline to the rhizome of *Arabidopsis* resulted in a reduced ROS level, suggesting the potential role of proline in ROS detoxification [7]. Other studies also reported positive correlations between exogenous proline application and enhanced antioxidant enzyme activity in chickpea, sugarcane, melon, etc. [8,9,10,11]. Along with its role in ROS detoxification, proline also plays a crucial role in protein modification and cell membrane stabilization [12,13]. Recently, other metabolic pathways, such as amines and γ-aminobutyric acid, were also reported to interact with proline metabolism under drought stress [14].

The substantial increase in proline content under drought stress reflects its enhanced biosynthesis and/or reduced degradation [4,15,16,17]. In plants, proline biosynthesis is initiated by Δ^1^-pyrroline-5-carboxylate synthase (P5CS), the rate-limiting enzyme that catalyzes two sequential reactions to convert L-glutamate into glutamate-γ-semialdehyde (GSA) [18]. Overexpression of P5CS has also been demonstrated to improve stress tolerance in different plants [19]. *Arabidopsis thaliana* has two P5CS isoforms, P5CS1 and P5CS2 (encoded by *At2G39800* and *At3G55610*, respectively). While P5CS1 has a chloroplast localization, P5CS2 localizes in the cytosol, but shifts to chloroplasts under abiotic stress [20]. P5CS1 is the main isoform for stress-induced proline synthesis [21,22,23].

ORANGE (OR) is a DnaJ-like zinc finger protein that plays important roles in regulating plastid metabolism and development [24]. With dual localizations in both the chloroplasts and nucleus, OR was demonstrated to regulate carotenoid biosynthesis in chloroplasts, through posttranscriptional regulation of phytoene synthase (PSY), a rate-limiting enzyme in carotenoid biosynthesis, as well as to repress chlorophyll biosynthesis and chloroplast biogenesis by suppressing the transcriptional activity of the transcription factor TCP14 in the nucleus [24,25,26]. Recently, OR and its homologs have frequently been reported to function in plant resistance to abiotic stresses through different mechanisms [13,27,28,29,30]. These proteins all share a characteristic C-terminal zinc finger domain, with tandemly arranged cysteine-rich signatures, and are classified as type E DnaJ chaperones [31]. Such a C-terminal structure may facilitate their functions as molecular chaperones in maintaining the structures and/or functions of their partners conferring their various functions.

In this study, we demonstrated that OR contributes to drought stress tolerance, through its function in enhancing P5CS activity for proline biosynthesis.

## 2. Results

### 2.1. OR Positively Regulates Drought Stress Tolerance

**Figure 1:**
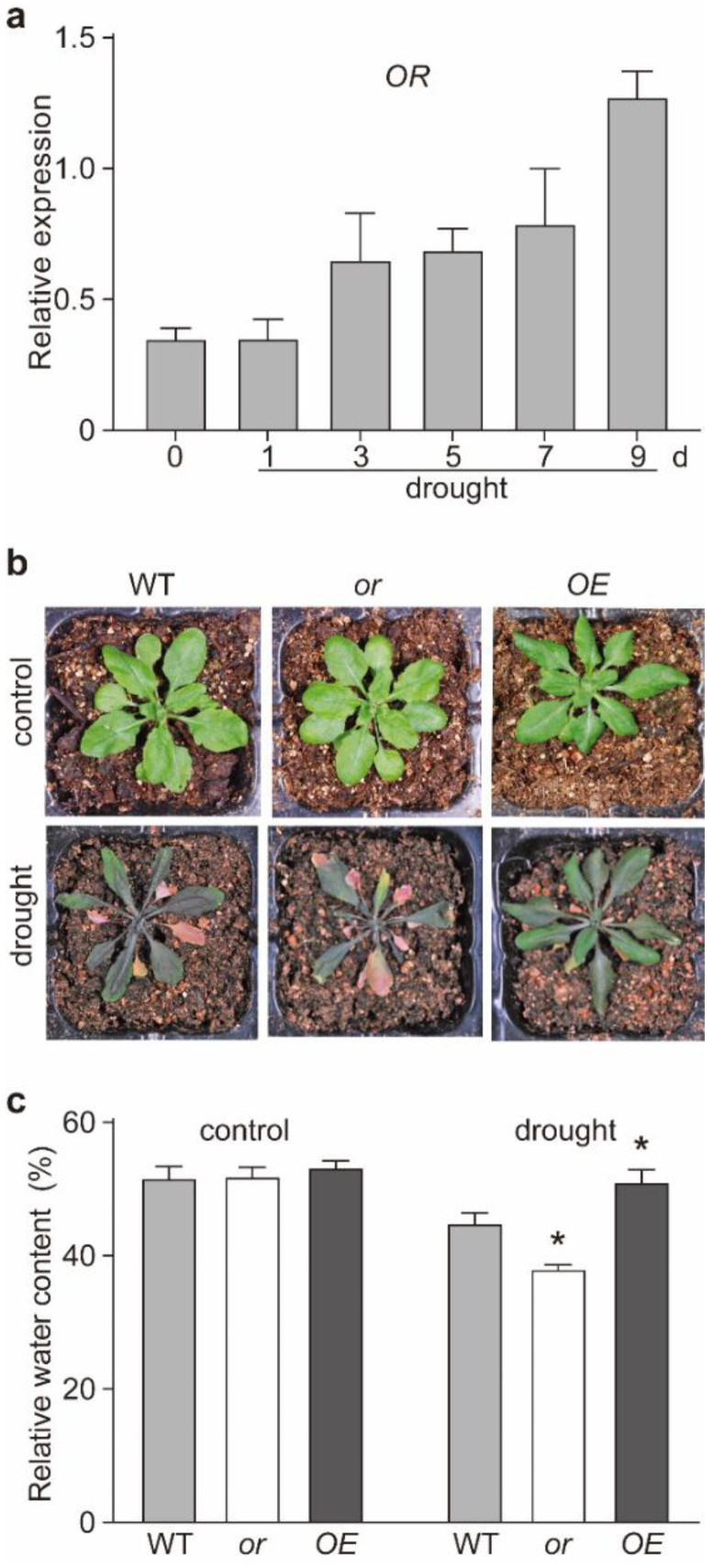
Response of different OR transgenic lines to drought stress. (**a**) Transcript abundance of OR in the wild-type (WT) *Arabidopsis thaliana* seedlings, before (0) and after different days of drought treatment. Transcript abundance was determined by qRT-PCR, with Actin2 as a reference. Data are means ± SEM (*n* = 3). (**b**) Representative seedlings of the WT and OR-overexpressing (OE) and -silencing (or) of *A. thaliana* seedlings under normal growth conditions (control) and drought treatment for 15 d. (**c**) Relative water contents in the WT, OE, and or seedlings under normal growth conditions (control) and drought treatment for 10 d. Data are means ± SEM (*n* = 3, * *p* < 0.05, Student’s *t* test).

To investigate whether OR is involved in plant response to drought stress, we initially determined the fluctuation of OR transcript abundance in the wild-type (WT) *A. thaliana* seedlings under drought stress. Our quantification indicated a gradual induction of OR expression, when irrigation was withheld (Figure 1a).

The drought-inducible OR expression inferred that transgenic lines with different OR backgrounds might respond differently to drought stress. Therefore, we used the homozygous OR-overexpressing (OE),T-DNA insertion (or) and RNA interference (RNAi) seedlings, which we reported previously [24,32,33] (Figure S1). After a 15-d drought treatment, the or seedlings displayed severe wilting and a retarded growth phenotype, whereas the OE seedlings showed improved growth, compared with the WT (Figure 1b and Figure S1). We further measured the relative water contents (RWCs) in these lines. No significant difference was found among these lines with regular irrigation (Figure 1c). However, among drought-stressed lines, the RWC of the OE seedlings was the highest, and that of the or seedlings was the lowest (Figure 1c). Additional OE and RNAi lines also showed similar phenotypes (Figures S1 and S2).

**Figure 2:**
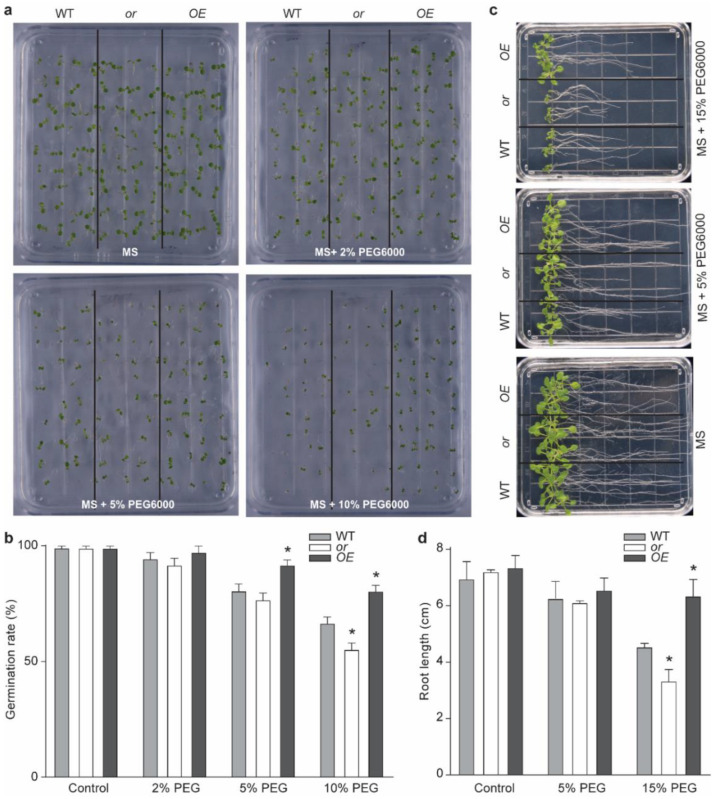
Response of different OR transgenic lines to PEG6000 treatment. (**a**) Germination of the wild-type (WT) and OR-overexpressing (OE) and -silencing (or) seeds on Murashige-Skoog (MS) plates, supplemented with indicated concentrations of PEG6000. (**b**) The germination rate of (**a**). Data are means ± SEM (*n* = 120. * *p* < 0.05, Student’s *t* test). (**c**) Growth of normally germinated seedlings on MS plates, supplemented with indicated concentrations of PEG6000. (**d**) Root length of different lines in (**c**). Data are means ± SEM (three plates with 15 seedlings were counted. * *p* < 0.05, Student’s *t* test).

We then studied whether OR transgenic seedlings had other related phenotypes. We tested the germination capacity of these lines on Murashige-Skoog (MS) plates, supplemented with different concentrations of PEG6000. No significant difference was observed among these lines, with different OR backgrounds on plates, containing 0% or 2% PEG6000 (Figure 2a,b). When 5% or 10% PEG6000 was supplemented, seeds of the OE line showed a significantly higher germination rate than the other two lines, whereas the germination rate of the or seeds was much lower than the WT and OE lines under 10% PEG6000 (Figure 2a,b). We further germinated the seeds of each of these lines on MS plates and moved the one-week-old seedlings to PEG6000-supplemented plates (Figure 2c). On MS plates containing 15% PEG6000, the root length of the OE seedlings was significantly longer than the other two lines, whereas that of the or seedlings was the shortest among all lines (Figure 2c,d).

**Figure 3:**
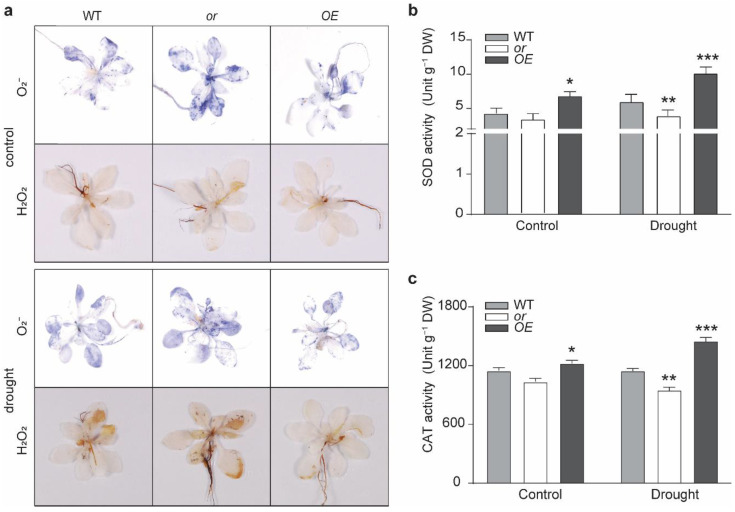
ROS production and antioxidative enzyme activities in OR transgenic seedlings. (**a**) Nitrotetrazolium blue chloride (NBT) and 3,3′-diaminobenzidine (DAB) staining showed the production of O_2_^−^ and H_2_O_2_ in the wild-type (WT) and OR-overexpressing (OE) and -silencing (or) seedlings under normal growth conditions (control) and drought treatment. (**b**,**c**) Enzymatic activity of superoxidase (SOD) (**b**) and catalase (CAT) (**c**) in different lines under normal growth conditions (control) and drought treatment. Data are means ± SEM (*n* = 3, * *p* < 0.05, ** *p* < 0.01, *** *p* < 0.001, or better, Student’s *t* test). DW, dry weight.

Because drought stress usually results in oxidative stress to plants, we stained the seedlings with 3,3′-diaminobenzidine (DAB) and nitrotetrazolium blue chloride (NBT), in order to assess their H_2_O_2_ and O_2_^−^ contents, respectively. Under normal growth conditions, similar staining intensities for each molecule were observed among these lines (Figure 3a). Drought stress induced the production of H_2_O_2_ and O_2_^−^ in all these lines, but at different levels. The production of both molecules was significantly alleviated in the OE seedlings, but was more severe in the or seedlings under the stressed condition (Figure 3a). We then determined the activities of superoxidase (SOD) and catalase (CAT), the two major enzymes responsible for scavenging ROS (as the first line of defense) [34,35,36]. Our results indicated remarkably enhanced SOD and CAT activities in the OE seedlings, under both control and drought-stressed conditions (Figure 3b,c). Activities of SOD and CAT in or seedlings were significantly lower than their corresponding WT levels under drought stress, but were at comparable levels with the WT under normal growth conditions (Figure 3b,c).

**Figure 4:**
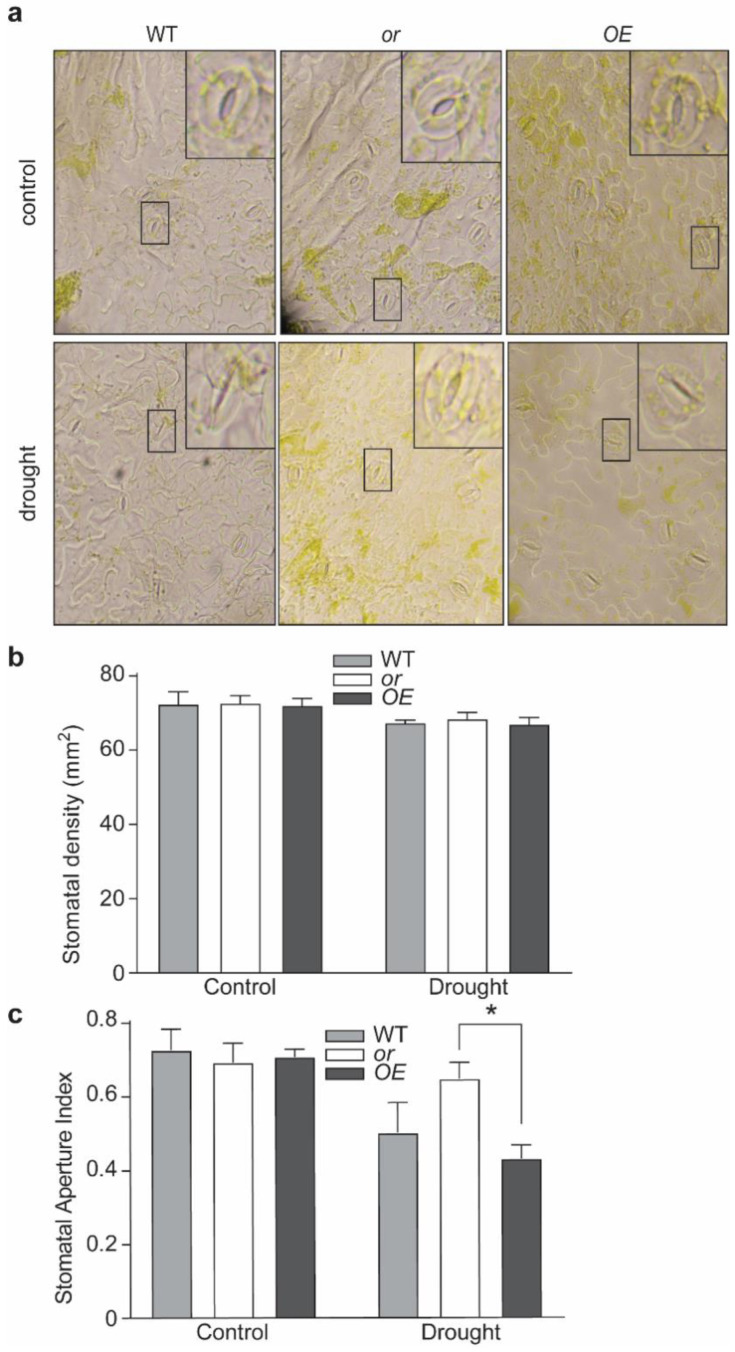
OR affects the response of stomatal apertures to drought stress. (**a**) Microscopic observation of stromata from the wild-type (WT) and OR-overexpressing (OE) and -silencing (or) seedlings under normal growth (control) and drought treatment. (**b**) Stomatal densities of the WT, OE, and or lines. Data are means ± SEM (*n* = 20). (**c**) Stomatal aperture index of different lines under normal growth conditions (control) and drought treatment. Data are means ± SEM (*n* = 15) * *p* < 0.05, Student’s *t* test).

We also compared the stomatal density and stomatal aperture index (SAI) on the abaxial leaf surface. Seedlings with different OR backgrounds showed no significant difference in their stomatal densities, under both control and drought-stressed conditions, indicating that OR does not modulate the biogenesis and/or development of stomata (Figure 4a,b). No SAI difference was observed among different lines under the control condition (Figure 4c). Drought treatment resulted in smaller stomatal apertures in all three lines, among which the or seedlings had the highest SAI (Figure 4c).

### 2.2. OR Modulates Proline Biosynthesis

**Figure 5:**
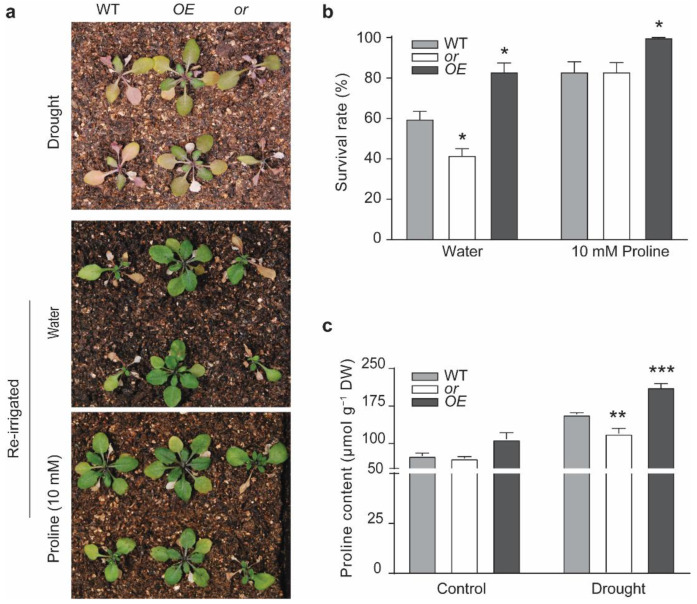
OR transgenic seedlings showed different proline levels under drought treatment. (**a**) Representative seedlings of the wild-type (WT) and OR-overexpressing (OE) and -silencing (or) lines under drought treatment and re-irrigated with water or 10 mM proline for 5 d. (**b**) Survival rate of water or proline re-irrigated WT, OE, and or seedlings. Data are means ± SEM (*n* = 9, * *p* < 0.05, Student’s *t* test). (**c**) Proline contents in drought or PEG6000 (15%)-treated WT, OE, and or seedlings. DW, dry weight. Data are means ± SEM (*n* = 3, * *p* < 0.05, ** *p* < 0.01, *** *p* < 0.001, or better, Student’s *t* test).

Proline is an important protective compound accumulated in plants under drought-induced oxidative stress [37]. To address whether the improved drought tolerance, by the overexpression of OR, was also related to proline, we treated seedlings of different lines under drought for 15 d, and then re-irrigated them with water or 10 mM proline (Figure 5). From our observation, the seedlings re-irrigated with water showed different survival rates, among which, the OE seedlings had the highest rate, and the or seedlings had the lowest (Figure 5a,b). However, when 10 mM proline was used for re-irrigation, both the WT and or seedlings showed improved survival rates, which were comparable with the OE seedlings re-irrigated with water (Figure 5b). The OE seedlings re-irrigated with 10 mM proline showed a significantly higher survival rate, compared with the other two lines (Figure 5b).

We then determined the contents of endogenous proline in the WT and OR transgenic lines. There was no significant difference among all the lines under normal growth conditions (Figure 5c). However, under drought stress, the or seedlings displayed a significantly lower level of proline, while the OE seedlings showed a substantially higher level of proline, both compared with the WT (Figure 5c).

**Figure 6:**
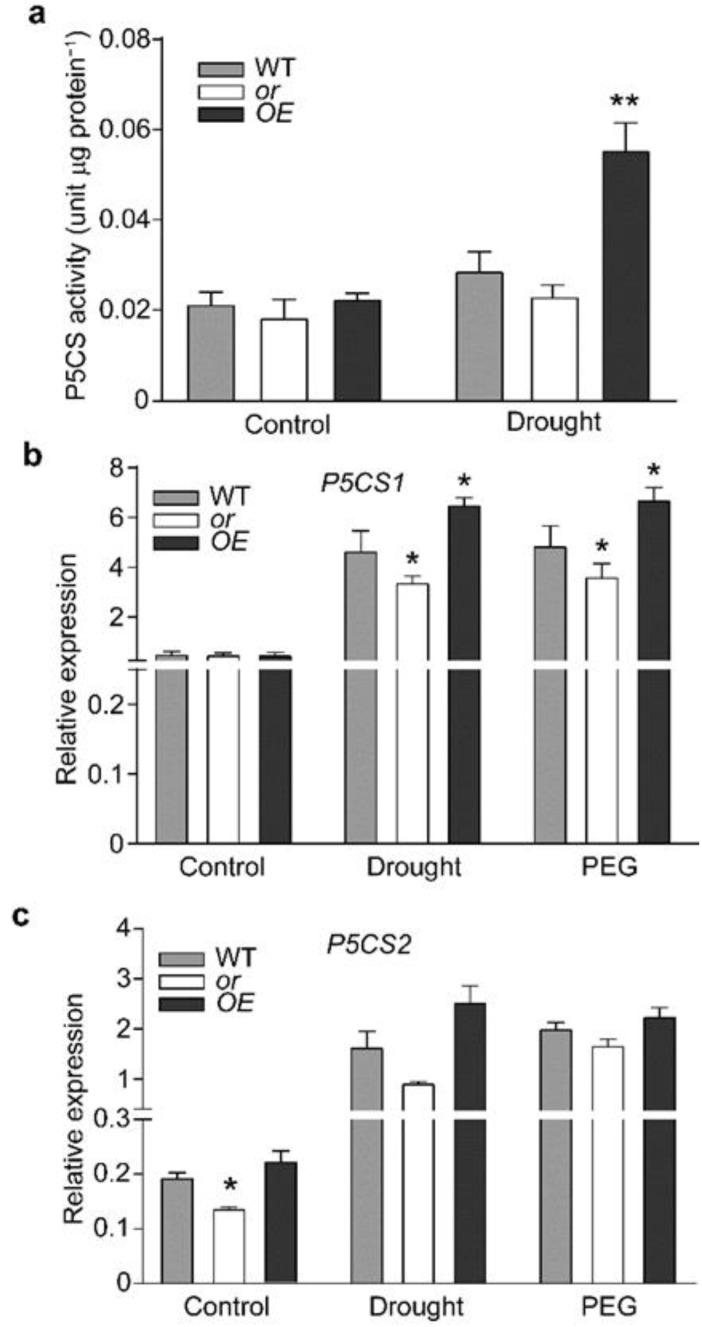
OR affects P5CS expression, in response to drought treatment. (**a**) P5CS activity in the wild-type (WT) and OR-overexpressing (OE) and -silencing (or) 4-week-old seedlings, under normal growth condition and drought treatment, for 7 d. (**b**,**c**) Transcript abundances of P5CS1 (**b**) and P5CS2 (**c**) in the WT, OE, and or seedlings, under normal growth conditions (control) or treated by drought or 10% PEG6000, for 7 d. Data are means ± SEM (*n* = 3, * *p* < 0.05, ** *p* < 0.01, or better, Student’s *t* test).

Because proline was found to highly accumulate in OE seedlings under drought stress, we postulated that its biosynthesis might be modulated by OR. To this end, we first determined the enzymatic activity of P5CS, the rate-limiting enzyme in the proline metabolic pathway, by testing the conversion from L-glutamate to γ-glutamyl phosphate. On a total protein basis, the P5CS activity was slightly higher in OE and lower in or seedlings, compared with the WT level under the control condition; although, the differences were not significant among these lines (Figure 6a). Drought stress significantly enhanced the enzymatic activity of P5CS in OE seedlings and induced that in WT seedlings at a marginal level (Figure 6a). However, the P5CS activities in or seedlings, under both control and drought treatment conditions, were similar (Figure 6a).

The enhanced P5CS activity in OE seedlings under drought stress could be a result of induced gene expression or/and improved enzymatic activity. We first quantified the transcript abundance of P5CS. *A. thaliana* has two genes for P5CS, i.e., P5CS1 and P5CS2. Our quantification showed that P5CS1 had an overall higher expression level than P5CS2 in different lines (Figure 6b,c). P5CS1 had lower expression levels in or seedlings and higher levels in OE seedlings under drought stress or PEG6000 treatment, compared with the corresponding WT levels (Figure 6b). Although P5CS2 showed a lower expression level in or seedlings under the control condition, its expression levels among different lines, under both drought and PEG6000 treatment, showed no significant difference.

### 2.3. OR Enhances P5CS Enzymatic Activity

**Figure 7:**
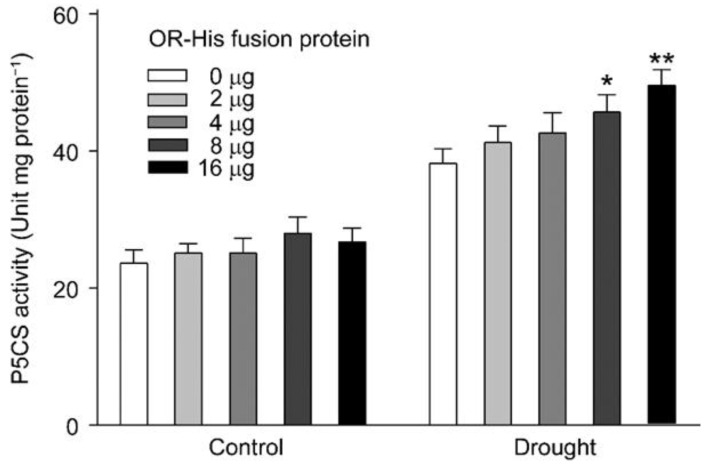
OR improves the enzymatic activity of P5CS. Total protein extract was prepared from the wild-type seedlings under normal growth conditions (control) or drought treatment. Different amounts of heterologously expressed and purified OR protein, with a His-tag (OR-His), was supplemented to the extract for P5CS enzymatic assay. Data are means ± SEM (*n* = 3, * *p* < 0.05, ** *p* < 0.01, or better, Student’s *t* test).

Because all three lines with different OR backgrounds showed similar transcript abundances of P5CS1, the major isoform in *A. thaliana*, under normal growth conditions, it did not seem like the expression of OR directly regulated P5CS1 expression. We then tried to figure out whether OR could modulate the catalytic activity of P5CS by an in vitro assay. In this assay, we heterologously expressed and purified OR with a His-tag (OR-His), and then supplemented this fusion protein at different amounts to aliquots of a total protein extract, prepared from the WT seedlings. When seedlings under normal growth conditions were used, the supplement of exogenous OR-His, at different levels, did not affect P5CS activity (Figure 7). However, when we used the seedlings under drought stress, P5CS enzymatic activity raised significantly with the increased OR-His level (Figure 7).

## 3. Discussion

With its dual subcellular localization, OR has been reported to regulate the biosynthesis of both carotenoids and chlorophylls, as well as the development of both chromoplasts and chloroplasts [24,26,32,38,39]. Other functions of OR, such as regulating petiole elongation and resistance to abiotic stresses, have also been reported [13,27,28,30,40]. In this study, several lines of evidence indicated that OR also contributes to drought tolerance and the production of the antioxidant compatible compound proline. First, we observed that the expression of OR was upregulated by drought treatment (Figure 1a). Second, transgenic seedlings overexpressing OR (OE) demonstrated a better tolerance to drought stress than the WT and OR-silencing (or) lines; although, no significant difference was detected among seedlings with different OR backgrounds grown under normal growth conditions. Drought-stressed OE seedlings retained a higher level of relative water content (Figure 1c and Figure S1) and possessed higher activities of ROS scavenging enzymes SOD and CAT, together with lower levels of both H_2_O_2_ and O_2_^−^ (Figure 3). Our germination assay also showed that OE seeds had a higher germination rate on MS plates supplemented with 5–10% PEG6000, and their roots were longer on MS plates with 15% PEG6000 (Figure 2). Third, OE seedlings were found to accumulate a relatively higher proline level, compared with the WT and or seedlings, under drought stress, and the re-irrigation with 10 mM proline was able to rescue the retarded growth of drought-stressed WT and or seedlings (Figure 5). These results suggest that OR is involved in the drought tolerance of *A. thaliana*.

It was interesting to find the differences in proline content and P5CS activity among these lines under drought stress. We initially tried to determine whether there was a transcriptional regulation. *A. thaliana* has two genes encoding P5CS, P5CS1, and P5CS2 (47). P5CS2 showed an overall much lower expression level than P5CS1 and did not respond to drought treatment (Figure 6). Different from P5CS2, the expression of P5CS1 was induced by both drought and PEG6000 treatments (Figure 6). Therefore, it seems possible that the relatively higher P5CS activity in drought-treated OE seedlings, compared with WT and or seedlings, was a result of the induced expression.

However, from our quantification, drought stress induced the expression of P5CS1 in all three lines, with different OR backgrounds, although at different levels (Figure 6b). This suggested that the differential expression of P5CS1 alone was not sufficient for the significantly higher P5CS activity in drought-stressed OE seedlings. To this end, we tried to figure out the contribution of OR to P5CS activity, because the only genetic difference among these lines was OR, the gene induced by drought stress. Our in vitro enzymatic assay confirmed our postulation. In this assay, we first determined P5CS activity using the total protein extract, prepared from non-stressed WT seedlings. No difference was found when the various amount of purified OR protein was added (Figure 7). Therefore, with a low level of P5CS in non-stressed seedlings, OR did not affect the conversion from L-glutamate to γ-glutamyl phosphate. This agreed with our quantification, which showed similar proline contents among these three lines, with different OR backgrounds, under normal growth conditions (Figure 5c). We then prepared total protein extract from drought-stressed WT seedlings, which had a higher level of P5CS activity, compared to the non-treated seedlings (Figure 6a and Figure 7). When OR was supplemented to this preparation, we observed an increase of P5CS activity with the rise of OR amount (Figure 7). This indicated that the supplement of exogenous OR favored the enzymatic activity of endogenous P5CS. Therefore, it is reasonable to conclude that the drought-induced expression of endogenous OR promoted the activity of P5CS, the key enzyme for proline biosynthesis. This explains the drought tolerance of the OE seedlings, as well.

Moreover, OR was also reported to have nucleus localization, where it interacts with transcription factors to modulate gene expression [24,32,33]. Separate transcription factors, such as MYC2, WRKY40, and bZIP53, etc., were previously reported to regulate proline metabolism [41,42,43,44]. To clarify whether OR also regulates drought tolerance through transcriptional modulation, we transformed *A. thaliana* WT seedlings to overexpress a truncated version of OR (OR^nuc^), which we previously demonstrated to specifically localize in the nucleus [32,33] (Figure S1). No distinct difference was observed when the WT, OE, and OR^nuc^ seedlings were grown under normal growth conditions (Figure S2). However, under drought stress, the growth of OR^nuc^ seedlings showed a wilting phenotype and retarded growth, similar to the WT, while that of the OE seedlings was rescued (Figure S2). Therefore, the nuclear localization of OR does not contribute to its function in drought stress tolerance.

## 4. Conclusions

In this study, we demonstrated that the DnaJ-like zinc finger protein OR functions in drought-stress tolerance through its promotion of the enzymatic activity of P5CS in the production of proline. This not only added a novel function of OR, besides its regulation of carotenoid and chlorophyll metabolism and plastid development, but also indicated the participation of chaperones in stress response. P5CS is not the first enzyme of which the activity is modulated by OR. OR was also reported to stabilize and promote the activity of phytoene synthase (PSY), the entry enzyme for carotenoid biosynthesis, through protein–protein interaction [26]. It would be very interesting to decipher the spatiotemporal pattern of the interaction between P5CS and OR and figure out whether other type E DnaJ chaperones, with the highly conserved C-terminal zinc finger domain, also have similar functions on different enzyme partners.

## 5. Materials and Methods

### 5.1. Plant Growth Conditions and the Treatments

For all experimental work, seedlings were with *Arabidopsis thaliana* Columbia-0 wild-type (Col-0 WT) background. The overexpression (OE#1, OE#2), T-DNA insertion (or), and RNAi lines of OR were from our previous studies [24,32,33]. The line overexpressing a nuclear-targeted truncation of OR (OR^nuc^) was also from our previous studies [17,30]. All the WT and transgenic lines have been self-crossed to homozygous in previous studies. In general, seeds were sterilized with 75% ethanol and planted on half-strength Murashige-Skoog (MS) medium, followed by stratification for 3 d at 4 °C in the dark. After stratification, the plates were shifted to a greenhouse, at 22 °C, under a 16 h/8 h light/dark regime, with an irradiance of 100 μmol photons m^−2^ s^−1^ [30]. Each experimental work was carried out in biological triplicates. For germination assay, different percentages (2, 5, and 10%) of PEG6000 were supplemented to the 1/2 MS plates [45]. The germination rate (%) was calculated from 120 seeds for each line.

The role of OR in drought stress response was performed either through water withholding irrigation or PEG6000 treatment. For water withholding experiments, the WT and OR transgenic lines, including OE and -silencing or lines, were grown on MS medium for 10 d and then shifted to soil (a mixture of peat moss, vermiculite, and perlite at 3:2:1) and left to grow under regular irrigation.

After 4 weeks, the plants were exposed to 15 d of drought stress by withholding irrigation. For the survival assay, 15 d of drought-stressed plants were consecutively re-irrigated with either water or 10 mM proline. Plants that retained healthy growth with green leaves were considered as having survived. We also examined the germination capacity and post-germination effect of PEG6000 on WT and OR transgenic seedlings. For seed germination capacity, all the studied lines were investigated on a simple or 2%, 5%, and 10% PEG6000 supplemented medium. For the post-germination effect, one-week-old newly germinated WT and OR transgenic, including OE and or seedling, were shifted to different PEG supplemented media, and their root length analysis was performed, using ImageJ (ver. 1.8.0_172, https://imagej.nih.gov/ij/ (accessed on 1 February 2022)), after 3 weeks of growth periods.

### 5.2. ROS Detection

Reactive oxygen species (ROS), such as superoxide (O_2_^−^) and hydrogen peroxide (H_2_O_2_), were detected using nitrotetrazolium blue chloride (NBT) and 3,3′-diaminobenzidine (DAB), respectively, as described [46]. Four-week-old WT and transgenic lines (or and OE) from both control and drought-stressed conditions were collected, dipped in NBT or DAB staining solution under dark, and incubated at 28 °C for 3 and 8 h, respectively. After the incubation, the plants were de-stained in 80% boiling ethanol for 30 min and then photographed.

### 5.3. SOD and CAT Activity Assay

For determining the activities of SOD and CAT, 0.5–1.0 g leaf tissue was frozen in liquid nitrogen and thoroughly ground in phosphate-buffered saline (pH 7.4). The resultant homogenate was centrifuged at 10,000× *g* for 10 min at 4 °C. CAT and SOD activities were quantified using corresponding kits (Beyotime, Shanghai, China), following the manufacturer’s instructions. Protein content from the extract was determined according to that described in [47].

### 5.4. Stomatal Aperture Analysis

Stomatal aperture analysis was performed according to that described in [48]. Three fully expanded leaves per treatment were detached and polished with transparent polish to the abaxial side. After being dried, the nail polish layer was peeled, with the help of a transparent squash tap, and stained with 1 µM rhodamine 6G. After staining, the image was taken, with the help of a light microscope, equipped with a CCD camera. Stomata aperture was analyzed using ImageJ.

### 5.5. RNA Extraction, cDNA Synthesis, and qRT-PCR

Total RNA was extracted using the RNAiso reagent (TaKaRa, Shiga, Japan), following the manufacturer’s instruction. Total RNA of 1 μg was reverse-transcribed using the PrimeScript 1st strand cDNA synthesis kit (TaKaRa), following the manufacturer’s instruction. Quantitative real-time PCR (qRT-PCR) was performed using SYBR Premix ExTaq II (TaKaRa) in a Thermal Cycler Dice Real-Time System TP800 (TaKaRa), according to the manufacturer’s instructions. Gene expression values were calculated according to the comparative *C*_T_ method, using Actin2 as a reference [49]. Each data point represents at least three independent biological samples, with three or more technical replicates. All primers used for this study are given in Table S1.

### 5.6. Proline Content Analysis

Proline contents were assessed according to that described in [50]. Approximately 0.1–0.2 g fresh plant tissues were frozen in liquid nitrogen and thoroughly ground in 5 mL of 3% sulfosalicylic acid. The homogenate was centrifuged at 4 °C, at 5000× *g*, for 10 min; additionally, 1 mL supernatant was combined with 1 mL ninhydrin solution (0.5 g dissolved in 12 mL glacial acetic acid and 8 mL 6 M phosphoric acid). The reaction mixture was incubated in a water bath at 100 °C for 1 h, followed by partitioning against 2 mL toluene. Absorbance at 520 nm was measured in the upper organic layer. A standard curve was performed using authentic L-proline.

### 5.7. Purification of OR-His Protein

C-terminal transit peptides (cTPs) of chloroplast proteins are usually excited after importing to generate mature proteins [51]. For producing the recombinant OR protein fused with a His-tag, the coding region for the truncated OR (Ala^56^-Asp^307^), without its cTP (OR^ΔcTP^), was amplified from a cDNA pool, prepared in our previous study, and cloned into pET32 (EMD Millipore, Billerica, MA, USA) [32]. The fusion protein was expressed in Rosetta2(DE3) cells (EMD Millipore) and purified by affinity chromatography using Ni-NTA magnetic beads (Sangon, Shanghai, China), according to the manufacturer’s instruction.

### 5.8. P5CS Enzymatic Assay

P5CS activity was examined as a γ-glutamyl kinase in the enzyme extract mixture that catalyzed the conversion of L-glutamate to γ-glutamyl phosphate, as previously described [52]. *Arabidopsis* seedlings (0.25 g) were ground in liquid nitrogen and homogenized in the extraction buffer containing 100 mM Tris-HCl, 10 mM β-mercaptoethanol, 10 mM MgCl_2_, 4 mM DDT, 1 mM EDTA, 2 mM PMSF, and 2% PVP (pH 7.5). The crude extract was centrifuged twice at 4 °C for 20 min, at 1000× *g*, and used as the total protein extract. The enzymatic assay was conducted in the assay buffer containing 50 mM Tris-HCl, 50 mM L-glutamate, 100 mM hydroxylamine-HCl, 20 mM MgCl_2_, 10 mM ATP, and the protein extract (or with additional OR-His fusion protein), in a total volume of 0.5 mL. The reaction was initiated by incubating the reaction mixture at 37 °C for 15 min and stopped by adding 1 mL stopped buffer, containing 2.5% FeCl_3_, 6% trichloroacetic acid in 2.5 N HCl.

The formation of γ-glutamyl phosphate was assessed through colorimetric determination at 535 nm, against a blank control, conducted in parallel, without ATP, in the reaction system. The enzyme activity was expressed in unit µg protein^−1^, which represents the amount of enzyme required to produce 1 µmol of γ-glutamyl hydroxamate per min [39]. Protein contents were determined, as reported in [47].

## Data Availability

Not applicable.

## Supplementary Materials

The following supporting information can be downloaded at: https://www.mdpi.com/article/10.3390/ijms23073907/s1.
