# Supplementary material for: The DnaJ-like Zinc Finger Protein ORANGE Promotes Proline Biosynthesis in Drought-Stressed Arabidopsis Seedlings
Source: Int J Mol Sci. 2022 Mar 31;23(7):3907. doi: 10.3390/ijms23073907 (PMC8999238; doi:10.3390/ijms23073907)
Supplement: Supplementary file 1 [file ijms-23-03907-s001.zip › ijms-1620124-supplementary/Table S1.pdf]

Table S1. Primers used in this study.

Primer	Sequence (5’-3’)
For qRT-PCR	
OR-qF	TTTGTCTGTTTCCTACCCA
OR-qR	GAATCGCCATCTGAGTCT
ACT2-qF	CCAACATATGCATCCTTCTGGTTCATCCCA
ACT2-qR	TGGCTGAGGCTGATGATATTCAACCAATCG
P5CS1-qF	CTATCCAGCAGCCTGTAATG
P5CS1-qR	CCGTGCTTCTGGTATGTTC
P5CS2-qF	GCATAGTGACGGAAGATAGTG
P5CS2-qR	TGAATCCTGCTTGTGCTTAT
For cloning OR-His	
OR-His-F	TCGAGCTCCGTCGACAAGCTTATGCCGATAAATTCGCTTCCG
OR-His-R	CTCGAGTGCGGCCGCAAGCTTATCGAAAGGGTCGATACG
