# Supplementary figures and images for: The DnaJ-like Zinc Finger Protein ORANGE Promotes Proline Biosynthesis in Drought-Stressed Arabidopsis Seedlings
Source: Int J Mol Sci. 2022 Mar 31;23(7):3907. doi: 10.3390/ijms23073907 (PMC8999238; doi:10.3390/ijms23073907)

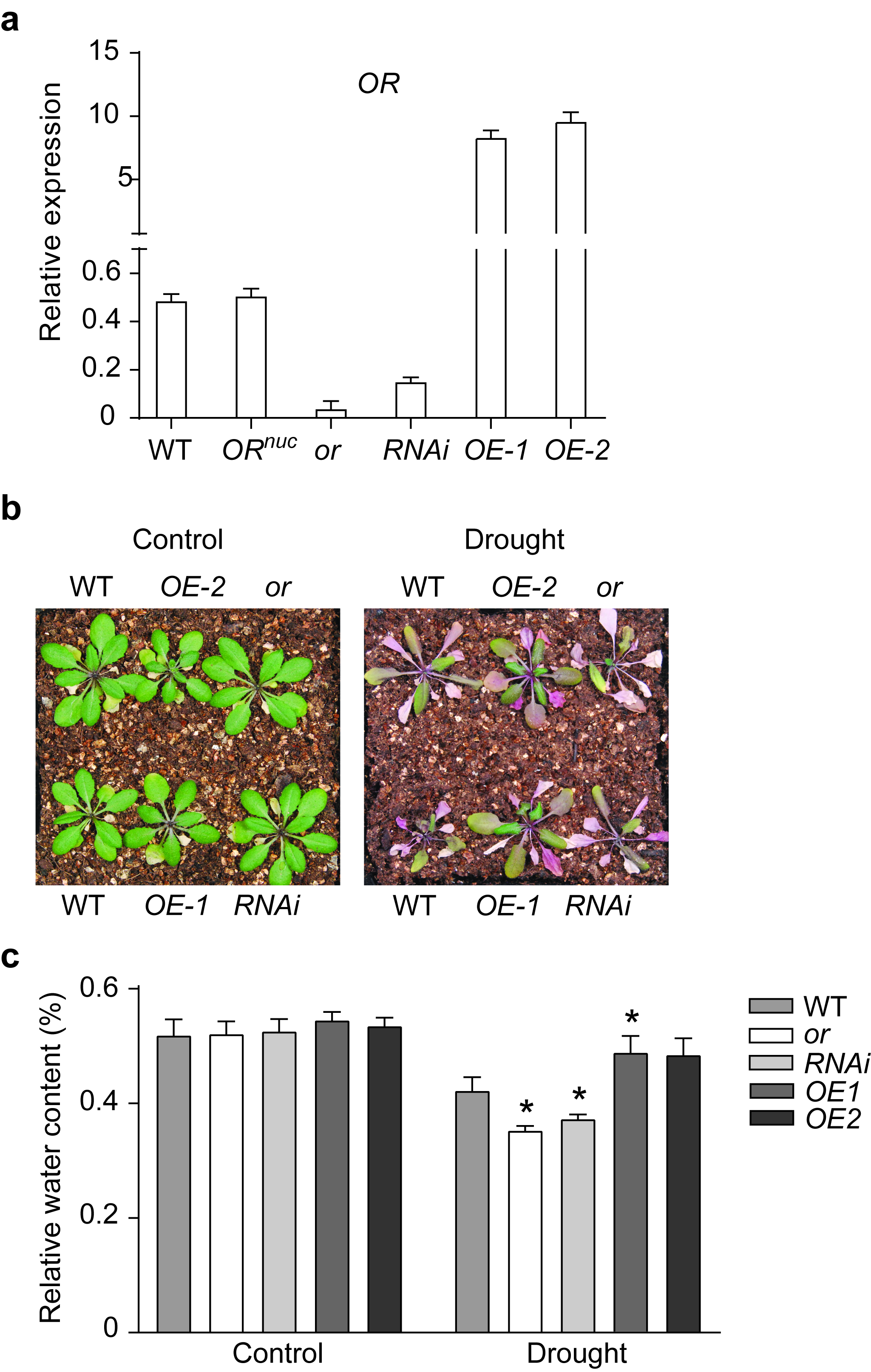

Supplement: Supplementary file 1 [file ijms-23-03907-s001.zip › ijms-1620124-supplementary/Figure S1-revised.tif]
